# Prevalence of HIV and syphilis infections among pregnant women attending antenatal clinics in Tanzania, 2011

**DOI:** 10.1186/s12889-015-1848-5

**Published:** 2015-05-22

**Authors:** Joel Manyahi, Boniphace S. Jullu, Mathias I. Abuya, James Juma, Joel Ndayongeje, Bonita Kilama, Veryeh Sambu, Josef Nondi, Bernard Rabiel, Geoffrey Somi, Mecky I. Matee

**Affiliations:** Muhimbili University of Health and Allied Sciences, Dar es Salaam, Tanzania; St. Francis University College of Health and Allied Sciences, Ifakara, Tanzania; National AIDS Control Programme, Dar es Salaam, Tanzania

**Keywords:** HIV, Syphilis, Pregnant women, Tanzania

## Abstract

**Background:**

The occurrence of HIV-1 and syphilis infections during pregnancy poses major health risks to the foetus due to mother-to-child transmission. We conducted surveillance of HIV and syphilis infections among pregnant women attending antenatal clinics (ANCs) in Mainland Tanzania in 2011.

**Methods:**

This surveillance was carried out in 133 ANCs selected from 21 regions in Tanzania. In each region, six ANC sites were selected, with urban, semi-urban, and rural areas contributing two each. All pregnant women who were attending selected sentinel ANC sites for the first time at any pregnancy between September and December 2011 were enrolled. Serial ELISA assays were performed to detect HIV infection in an unlinked anonymous manner using dried blood spot (DBS) after routine syphilis testing. Data analysis was conducted using Stata v.12 software.

**Results:**

A total of 39,698 pregnant women representing 2.4 % of all pregnant women (1.68 million) attending ANCs in the Mainland Tanzania were enrolled. The overall HIV prevalence was found to be 5.6 % (95 % CI: 5.4–5.8 %). The risk for HIV infection was significantly higher among women aged 25–34 (cOR = 1.97, 95 % CI: 1.79–2.16; *p* < 0.05), older than 35 years (cOR = 1.88, 95 % CI: 1.62–2.17; *p* < 0.05) and those having 1–2 and 3–4 previous pregnancies. HIV infection was less prevalent among women attending rural ANC clinics (cOR = 0.46, 95 % CI 0.4–0.52; *p* < 0.05).

The overall syphilis prevalence was 2.5 % (95 % CI: 2.3, 3.6). The risk for syphilis infection was significantly higher among women attending semi-urban and rural clinics and those having 3–4, and 5 previous pregnancies (*p* < 0.05). Marital status and level of education were not statistically significant with either of the two infections. HIV and syphilis co-infections occurred in 109 of 38,928 (0.3 %).

**Conclusion:**

The overall prevalence of HIV infection (5.6 %) and syphilis (2.5 %) found among pregnant women attending ANC clinics in Tanzania calls for further strengthening of current intervention measures, which include scaling up the integration of prevention of mother to child transmission (PMTCT) services in Reproductive and Child Health (RCH) clinics.

## Background

In many developing countries, including Tanzania, estimates on the magnitude of and trends on the HIV epidemic are obtained through HIV seroprevalence surveys conducted over a period of time [1]. These surveys are primarily conducted using sentinel and general population. HIV seroprevalence targeting the general population allows more accurate estimation of the national HIV prevalence than traditional sentinel surveillance, and have been of useful value in assessing the scale of epidemics worldwide [2–4]. Despite that sentinel surveillance remains the main instrument in estimating HIV prevalence in many developing countries due to financial and logistical reasons. In Tanzania the most frequently used sentinel populations are women attending antenatal clinics (ANCs) and persons attending clinics for routine diagnosis and treatment of sexually transmitted infections (STIs) [5–7]. Apart from these surveys, the country has also conducted household surveys to monitor HIV infection and behavioral risks factors in general population [8–10]. Results from various surveys have reported varying trends of both HIV and other sexual transmission diseases [8, 11, 12].

In Tanzania the last round of ANC HIV and syphilis surveillance was conducted in 2007 (reference). This has left a gap regarding the dynamics of HIV and syphilis infections among pregnant women attending ANC clinics in Tanzania. Therefore, in 2011 the National AIDS Control Programme (NACP) decided to conduct a country-wide surveillance to estimate the prevalence of HIV and syphilis among pregnant women attending ANC in Tanzania. The ultimate goal was to provide important information for program planning and evaluation of various interventions taken by the Government of the United Republic of Tanzania and other stakeholders, including development partners.

## Methods

### Selection of surveillance sites

A total of 133 ANC (44 health centres, 48 dispensaries, 24 hospitals and 17 reproductive and child health clinics) were selected from all 21 regions of mainland Tanzania. In most regions two sites were purposively selected from urban, semi-urban and rural settings except for Dar es Salaam (the capital city), in which all the selected sites were classified as urban. ANC sites were considered for selection if they could enroll at least 120 pregnant women during the three consecutive months of data collection. In total, 46 sites were located in urban (city and/or town at the regional headquarters), 40 in semi-urban (district headquarters, country borders, or towns situated along major roads), and 47 in rural (remote areas where communities either farm or keep livestock) areas.

### Surveillance target population

The surveillance population included all pregnant women of all ages attending a selected sentinel ANC site for the first time at any pregnancy during survey period between September and December 2011.

### Specimen collection and preparation for testing

About 3–5 ml of whole blood was collected from each woman in an Ethylenediamine Tetra acetate (EDTA) vacutainer tube for routine syphilis testing. In addition, a drop of blood (≈100 μl) was used to prepare DBS specimens for HIV surveillance purposes. The DBS cards were left overnight to dry at room temperature. The same surveillance number was also written on the surveillance data collection form. At this point, the upper part of the surveillance data collection form that contained the woman’s clinic card number was torn up and discarded in order to ensure de-linking of the client identifier from the respective HIV data that was documented on the surveillance data collection form.

### Storage and transportation of DBS

Dried DBS cards were stacked between weighing paper and stored in zip-locked plastic bags with desiccant (drying) packets and a humidity indicator card together with their completed data collection forms. Desiccant packs were changed when humidity indicator cards changed color from blue to pink. On weekly basis using courier service, ANC survey staff mailed completed data collection forms and DBS samples to their assigned zonal laboratory. From zonal laboratory specimens were transported to the Department of Microbiology and Immunology at Muhimbili University of Health and Allied Sciences (MUHAS) for testing.

### Social-demographic information

Surveillance staff recorded social and demographic details of the women at the site. The recorded information included age, marital status, parity, educational level and duration of stay at present residence.

### Syphilis testing and treatment

Testing for syphilis infection was done on site using rapid plasma reagin (RPR), which is a routine test for syphilis infection among ANC attendees in Tanzania. In the majority of rural sites nurses at ANC clinics performed the test, whereas in most of the urban and semi-urban sites laboratory staff performed tests. Results were recorded directly on the data collection form and on the woman’s clinic card or laboratory investigation request form. Women whose RPR test results positive were offered treatment based on the National sexual transmitted infections (STIs) Treatment Guidelines [13].

### HIV testing and quality assurance

The Department of Microbiology and Immunology at Muhimbili University of Health and Allied Sciences (MUHAS) collaborated with the Centers for Disease Control and Prevention (CDC) in developing a DBS HIV testing protocol and algorithm. At the laboratory, the DBS cards were eluted and tested for the presence of IgG antibodies to HIV using screening test, Vironostika® HIV Uni-Form II Ag/Ab ELISA test (Biomerieux, The Netherlands). Specimens with negative results underwent no further testing and were considered negative. Reactive samples underwent a second ELISA test, Enzygnostic Intergral II (Siemens Health care Diagnostic Products, GMBH, Marbug-Germany). Specimens that were reactive on both ELISA tests were considered HIV antibody positive. All discordant specimens were sent to the Tanzania’s National Health Laboratory Quality Assurance and Training Center (NHLQATC) for resolution.

### Ethical considerations

Because syphilis screening is already routinely conducted as per the guidelines of the Ministry of Health and Social Welfare (MOHSW) and HIV testing was performed on non-linked samples, informed consent was not warranted. There were no direct benefits or risks to participating in the survey, and data was de-identified before analysis to protect client confidentiality. Before actual protocol implementation, the National Institute for Medical Research (NIMR) approved the ANC surveillance protocol (reference number NIMR/HQ/R.8C/Vol 1/43).

### Data analysis

Data analysis was performed using Stata v.12 software (Stata Corporation, College Station, Texas, USA). Association between HIV and syphilis infections with variables were determined through univariate and multivariate logistic regression and expressed through odds ratio and 95 % confidence interval. Factors that were found to be significant in the univariate logistic regression analysis were used in the multivariate logistic regression and a *p*-value less than 0.05 was considered to be significant.

## Results

### Distribution of ANC attendees by region and locality

A total of 39,698 pregnant women, equivalent to 2.4 % of estimated 1.68 million women attending ANC in Mainland Tanzania were enrolled. The number of women enrolled varied by region, ranging from 867 in Mtwara to 3780 in Dar es Salaam (Table 1), while the percentage of those enrolled ranged from 1.2 % in Kagera to 5.1 % in Arusha.

**Table 1:** The 2011 ANC attendees, estimated number of pregnant women and percentage enrolment, Tanzania

Region	ANC attendees	**Estimated pregnant women	% Enrolled
Arusha	2812	55,551	5.1 %
Coast	1848	37,507	4.9 %
Dar es Salaam	3780	96,812	3.9 %
Dodoma	1789	77,580	2.3 %
Iringa	1221	50,667	2.4 %
Kagera	1533	125,594	1.2 %
Kigoma	1293	92,377	1.4 %
Kilimanjaro	1269	43,087	2.9 %
Lindi	1071	27,696	3.9 %
Manyara	1879	61,609	3.0 %
Mara	1255	88,145	1.4 %
Mbeya	2563	116,594	2.2 %
Morogoro	1576	70,087	2.2 %
Mtwara	867	45,159	1.9 %
Mwanza	2969	136,739	2.2 %
Rukwa	2033	71,855	2.8 %
Ruvuma	1651	50,986	3.2 %
Shinyanga	3385	190,957	1.8 %
Singida	1429	47,638	3.0 %
Tabora	1877	114,478	1.6 %
Tanga	1598	62,087	2.6 %
National	39,698	1,663,203	2.4 %

**Estimated number of pregnant women attended ANC clinic in 2011

### HIV prevalence in association with socio-demographic characteristics of the ANC attendees

Among 39,698 pregnant women enrolled in the surveillance, majority (87 %) were married and nearly half (48.7 %) were aged between 15–24 years. About 41.3 % had 1–2 previous pregnancies and most women (51.4 %) were from urban areas, and over two thirds (68.6 %) had primary education.

The overall prevalence of HIV was 5.6 % (95 % CI: 5.4, 5.8). Single women (6.8 %) were at more risk of being HIV infected than married women (5.4 %) (cOR = 1.28, 95 % CI: 1.13–1.45; *p* < 0.05) and divorced women (5.1 %) (Table 2). Women aged 25–34 (cOR = 1.97, 95 % CI: 1.79–2.16; *p* < 0.05) and those older than 35 (cOR = 1.88, 95 % CI: 1.62–2.17; *p* < 0.05) had increased risk of being HIV infected compared to age group 15–24 years. Women with 1–2 previous pregnancies (cOR = 1.94, 95 % CI 1.71–2.19); *p* < 0.05) and those with 3–4 previous pregnancies (cOR = 2.19, 95 % CI 1.92–2.59; *p* < 0.05) had significantly greater likelihood of being HIV infected compared to those who had more than 5 pregnancies (cOR = 1.23, 95 % CI 1.02–1.48); *p* = 0.07).

**Table 2:** Socio-demographical characteristics of the ANC attendees in association with HIV infection in Tanzania 2011

Variables	Number	HIV Prevalence (%)	cOR (95 % CI)	*P*-value	adOR (95 % CI)	*P*-value adOR
Marital status						
Married	34,543	5.4	1		1	
Single	4565	6.8	1.28(1.13–1.45)	<0.05	1.76(1.55–2.02)	<0.05
Divorced	78	5.1	1.67(0.84–3.32)	0.14	1.00(0.36–2.77)	0.99
Other	103	8.7	0.95(0.35–2.59)	0.91	1.84(0.92–3.68)	0.09
Age group						
15–24	19,316	3.9	1		1	
25–34	16,321	7.3	1.97(1.791–2.16)	<0.05	1.90(1.70–2.13)	<0.05
35+	3725	7.0	1.88(1.62–2.17)	<0.05	2.30(1.93–2.76)	<0.05
Not stated	336	4.1	1.08(0.63–1.85)	0.77	1.03(0.59–1.82)	0.90
Previous pregnancy						
0	10,487	3.45	1		1	
1–2	16,401	6.48	1.94(1.71–2.19)	<0.05	1.67(1.45–1.91)	<0.05
3–4	8121	7.25	2.19(1.92–2.59)	<0.05	1.59(1.35–1.89)	<0.05
>5	4004	4.2	1.23(1.018–1.48)	<0.05	0.88(0.69–1.11)	0.291
Not stated	694	4.9	1.44(1.05–2.07)	<0.07	1.25(0.86–1.82)	0.23
Residence						
Urban	20,397	6.59	1		1	
Semi Urban	10,680	5.82	0.88(0.79–0.96)	<0.05	0.90(0.82–0.99)	<0.05
Rural	8621	3.13	0.46(0.40–0.52)	<0.05	0.48(0.42–0.56)	<0.05
Education						
No formal education	6017	4.5	1		1	
Adult education	255	3.9	0.75(033–1.74)	0.51	1.22(0.44–3.38)	0.71
Primary education	27,222	6	0.65(0.23–1.85)	0.42	0.99(0.29–3.23)	0.99
Secondary	5521	4.9	1.02(0.45–2.34)	0.95	1.29(0.47–3.58)	0.62
Post secondary	581	2.9	0.83(0.36–1.91)	0.66	1.04(0.37–2.89)	0.94
Not stated	102	5.9	0.48(0.19–1.25)	0.14	0.55(0.18–1.68)	0.29
Duration of stay						
Less than 6 months	4991	5.3	1		1	
6 months and above	33,985	5.5	0.81(0.59–1.12)	0.20	0.88(0.63–1.22)	0.45
Not stated	722	6.5	0.85(0.63–1.15)	0.29	0.82(0.60–1.11)	0.19

*cOR* crude odd ratio, *aOR* adjusted odd ratio, *CI* confidence interval

The likelihood of being HIV infected was significantly less frequent among women residing in rural areas (3.13 %) (cOR = 0.46, 95 % CI 0.4–0.52; *p* < 0.05) and semi-urban areas (5.82 %) (cOR = 0.88, 95 % CI 0.8–0.96, *p* < 0.05) compared to urban areas (6.59 %). There was no statistically significant difference in the risk of HIV with education and duration of stay in residence.

On performing multivariate analysis after adjusting for potential confounding, the risk of HIV infection remained significantly high in the 25–34 year-old age group (aOR = 1.90, 95 % CI 1.70–2.13: *P* < 0.05) and in women older than 35 years (aOR = 2.30, 95 % CI 1.93–2.76; *p* < 0.05 compared to 15–24 years. Other independent factors of HIV infection were being single and having 1–2 (aOR = 1.50, 95 % CI 1.17–1.92; *P* < 0.05) and 3–4 previous pregnancies (aOR = 1.7, 95 % 1.27–2.29; *p* < 0.05) (Table 2), while those attending rural ANC clinics had a decreased risk of HIV infection (adOR = 0.48, 95 % CI 0.42–0.56; *p* < 0.05).

### Syphilis prevalence in association with demographic characteristics of ANC attendees

The overall syphilis prevalence was 2.5 % (956/38,920) (95 % CI: 2.3–3.6). The risk of syphilis infection was higher among women having 3–4 pregnancies (cOR = 1.94, 95 % CI: 1.60–2.35; *p* < 0.05) and >5 pregnancies (cOR = 2.48; 95 % CI 2–3.08; *p* < 0.05) as compared to 1–2 previous pregnancies (cOR = 1.23, 95 % CI 1.03–1.48; *p* = 0.06). Other characteristics significantly associated with syphilis were women attending semi-urban (3.1 %) (cOR = 1.88, 95 % CI 1.62–2.19; *p* < 0.05) and rural clinics (3.2 %) (cOR = 1.9, 95 % CI 1.61–2.24; *p* < 0.05). There was no association between syphilis infection and either age group, marital status, level of education or duration of stay in residence in a particular residence (Table 3).

**Table 3:** Prevalence of syphilis in association with social-demographic characteristics of the ANC attendees, Tanzania 2011

Variables	Number	Syphilis Prevalence	cOR (95 % CI)	*P*-value	adOR (95 % CI)	*P*-value adOR
Marital status						
Married	34,543	2.5	1		1	
Single	4565	2.2	0.90(0.733–1.12)	0.35	1.19(0.95–1.48)	0.12
Divorced	78	4	1.64(0.52–5.22)	0.40	1.47(0.458–4.69)	0.52
Other	103	1.9	0.78(0.19–3.2)	0.74	0.8(0.19–3.28)	0.76
Age group						
15–24	19,316	2.4	1		1	
25–34	16,321	2.8	1.37(1.19–1.57)	<0.05	1.12(0.94–1.33)	0.20
35+	3725	3.3	1.66(1.34–2.03)	<0.05	1.06(0.81–1.39)	0.67
Not stated	336	1.6	0.77(0.31–1.86)	0.56	0.76(0.31–1.86)	0.55
Previous pregnancy						
0	10,487	1.7	1		1	
1–2	16,401	2.1	1.23(1.03–1.48)	0.06	1.13(0.93–1.39)	0.23
3–4	8121	3.3	1.94(1.60–2.35)	<0.05	1.50(1.17–1.92)	<0.05
>5	4004	4.2	2.48(2.00–3.08)	<0.05	1.70(1.27–2.29)	< 0.05
Not stated	694	1.6	0.95(0.52–1.76)	0.88	0.78(0.41–1.49)	0.46
Residence						
Urban	20,397	1.69	1		1	
Semi Urban	10,680	3.13	1.88(1.62–2.19)	< 0.05	1.65(1.40–1.93)	< 0.05
Rural	8621	3.2	1.89(1.61–2.24)	< 0.05	1.37(1.14–1.65)	<0.05
Education						
No Formal Education	6017	4.5	1		1	
Adult Education	255	3.2	1.40(0.44–4.46)	0.57	1.02(0.32–3.29)	0.97
Primary education	27,222	2.3	0.96(0.25–3.71)	0.96	0.73(0.19–2.85)	0.65
Secondary	5521	1.3	0.69(0.22–2.17)	0.52	0.58(0.18–1.87)	0.37
Post Secondary	581	0.7	0.39(0.12–1.28)	0.12	0.39(0.12–1.26)	0.12
Not stated	102	3.3	0.21(0.05–0.96)	0.04	0.22(0.05–0.99)	0.05
Duration of stay						
Less than 6 months	4991	2.2	1		1	
6 months and above	33,985	2.5	0.79(0.48–1.29)	0.34	1.01(0.61–1.67)	0.97
Not stated	722	2.8	0.89(0.56–1.42)	0.63	0.87(0.55–1.38)	0.56

*cOR* crude odd ratio, *aOR* adjusted odd ratio, *CI* confidence interval

After adjusting for potential confounding, the likelihood of syphilis remained significantly higher among women having more than three previous pregnancies, and those who were attending semi-urban and rural ANC clinics.

### HIV and syphilis prevalence by location of ANC attendees

The prevalence of HIV infection ranged from 1.3 % (95 % CI: 1.0, 2.0) in Kigoma region to 14.8 % (95 % CI: 12.8, 16.8) in Iringa region (Table 4), whereas syphilis infection ranged from 0.4 % in Arusha to 6.7 % in Mwanza. In almost all regions, the 2011 HIV prevalence among ANC attendees is comparable to that of women from 2011/2012THMIS with minor variations, and both were higher than that of men and close to the general HIV prevalence (including men and women aged 15–49 years), Table 5.

**Table 4:** Prevalence of HIV and syphilis infection by region, Tanzania 2011

Region	HIV					SYPHILIS				
	Total	Positive	Prevalence (%)	95 % CI		Total	Positive	Prevalence (%)	95 % CI	
Arusha	2812	80	2.84	2.2	3.5	2719	10	0.37	0.1	0.6
Coast	1848	131	7.09	6	8.3	1845	62	3.36	2.5	4.2
Dar es salaam	3780	230	6.08	5.3	6.8	3633	44	1.21	0.9	1.6
Dodoma	1789	35	1.96	1.3	2.6	1769	63	3.56	2.7	4.4
Iringa	1221	181	14.82	12.8	16.8	1206	23	1.91	1.1	2.7
Kagera	1533	70	4.57	3.5	5.6	1488	70	4.7	3.6	5.8
Kigoma	1293	17	1.31	1	2	1242	9	0.71	0.2	1.2
Kilimanjaro	1269	40	3.15	2.2	4.1	1242	9	0.72	0.3	1.2
Lindi	1071	58	5.42	4.1	6.8	1031	18	1.75	0.9	2.5
Manyara	1879	45	2.39	1.7	3.1	1838	23	1.25	0.7	1.8
Mara	1255	53	4.22	3.1	5.3	1232	23	1.87	1.1	2.6
Mbeya	2563	290	11.31	10.1	12.5	2483	55	2.22	1.6	2.8
Morogoro	1576	88	5.58	4.4	6.7	1570	12	0.76	0.3	1.2
Mtwara	867	48	5.54	4	7.1	859	35	4.07	2.7	5.4
Mwanza	2969	126	4.24	3.5	5	2942	197	6.7	5.8	7.6
Rukwa	2033	170	8.36	7.2	9.6	2007	70	3.49	2.7	4.3
Ruvuma	1651	122	7.39	6.1	8.7	1633	45	2.75	2	3.6
Shinyanga	3385	195	5.76	5	6.5	3360	80	2.38	1.9	2.9
Singida	1429	70	4.9	3.8	6	1398	18	1.29	0.7	1.9
Tabora	1877	89	4.74	3.8	5.7	1824	56	3.07	2.3	3.9
Tanga	1598	76	4.76	3.7	5.8	1582	34	2.15	1.4	2.9
Total	39,698	2214	5.58	5.35	5.80	38,920	956	2.46	2.3	3.6

**Table 5:** Comparison of HIV prevalence according to 2011 ANC and 2011/12 THMIS data by region, Tanzania 2011/12

Region	2011 ANC HIV prevalence			2011–12 THMIS HIV prevalence*					
	Total	Positive	Prevalence (%)	Women		Men		Total	
				Prevalence	Number	Prevalence	Number	Prevalence	Number
Arusha	2812	80	2.84	3.9	290	2.3	245	3.2	535
Coast	1848	131	7.09	9.2	187	2.1	159	5.9	346
Dar es salaam	3780	230	6.08	8.2	962	5.3	802	6.9	1764
Dodoma	1789	35	1.96	2.1	373	3.7	332	2.7	705
Iringa	1221	181	14.82	10.9	183	6.9	145	9.1	328
Kagera	1533	70	4.57	5.5	399	4.1	355	4.8	754
Kigoma	1293	17	1.31	4.5	405	2.0	305	3.4	270
Kilimanjaro	1269	40	3.15	4.9	343	2.2	244	3.8	587
Lindi	1071	58	5.42	4.3	167	1.1	123	2.9	290
Manyara	1879	45	2.39	2.7	232	0.3	227	1.5	459
Mara	1255	53	4.22	5.2	385	3.5	321	4.5	706
Mbeya	2563	290	11.31	11.0	619	6.7	538	9.0	1157
Morogoro	1576	88	5.58	5.3	352	2.1	322	3.8	674
Mtwara	867	48	5.54	6.0	333	1.5	237	4.1	570
Mwanza	2969	126	4.24	4.7	509	3.7	411	4.2	920
Rukwa	2033	170	8.36	6.8	164	5.5	131	6.2	295
Ruvuma	1651	122	7.39	9.1	619	4.1	441	7.1	1061
Shinyanga	3385	195	5.76	8.1	368	6.6	313	7.4	681
Singida	1429	70	4.9	4.5	370	1.8	320	3.3	690
Tabora	1877	89	4.74	5.8	383	4.5	390	5.1	774
Tanga	1598	76	4.76	3.5	508	0.7	325	2.4	833
Total	39,698	2214	5.58	6.2	9756	3.8	7989	5.1	17, 745

*95 % CI for THMS were not available

Apparently, there was no clear association in the occurrence of the two infections. For example, while the prevalence of HIV infection in Iringa region was 14.82 % that of syphilis was only 1.91 %. Similarly, the prevalence of HIV infection in Mbeya region was 11.31 %, while that of syphilis was 2.2 %.

## Discussion

### Overall prevalence of HIV and syphilis infections

The 2011 ANC surveillance results shows that the overall prevalence of HIV infection was 5.6 % (95 % CI: 5.4, 5.8 %) while that of syphilis was 2.5 % (95 % CI: 2.3, 3.6 %). The observed HIV prevalence in this study was lower than the previous 2007/08 Tanzania ANC surveillance data (7 %) [14] and 2011/2012 Tanzania HIV-AIDS malaria indicator survey (THMS) (6 %) Table 5 [9]. With regard to the prevalence of HIV there was regional variations ranging from 1.3 % (95 % CI: 1.0, 2.0) in Kigoma to 14.8 % (95 % CI: 12.8, 16.8) in Iringa. The regional variations in HIV prevalence are in keeping with the previous Tanzania ANC surveillance data [8, 11, 14, 15] and a recent THMS survey [9] (Table 5). The NACP needs to investigate the possible reasons for persistently high prevalence of HIV infection in these regions. Our survey also showed HIV prevalence varied by residence with 3.1 %, 5.8 % and 6.6 % for rural, semi-urban and urban clinic attendees respectively, an observation which is in consistent with previous and recent reports from Tanzania [11, 13, 16]. These observed differences are probably due to differences in social-economic activities and level of wealth in urban versus semi-urban and rural areas [17, 18] as well as round sexual networks and prevalence of sex work in urban and semi-urban areas.

### Factors associated with HIV infection

In this survey, the risk of HIV infection significantly increased with age above 25 years, being highest (7.3 %) in women aged 25–34 years, followed by 35 years and above (7 %); finding which is in agreement with previous studies in Tanzania [11, 12], and elsewhere in Africa [19]. We also found relatively low risk of HIV infection among young women with no history of previous pregnancies compare to those 1–4 previous pregnancies (Table 2). The relatively low HIV prevalence (3.9 %) among women aged between 15–24years may signify a reduction in the number of new infections [20], however this need to be substantiated by trend analysis studies.

Women who reported single marital status (6.8 %) had significantly increased risk of HIV as compared to married women (5.4 %), an observation which is in keeping with the previous studies and ANC surveillance reports [11, 14, 15, 21]. Other reports in Tanzania have found highest HIV prevalence among married women or formerly married women [8, 13]. Such variations may imply that marital status per se is not an indicator of sexual activity and hence risk for HIV infection, which underlines the need to involve men in the control of HIV infection [22].

Notably, the risk of HIV infection decreased significantly with being in rural area as compared to semi-urban and urban areas is consistent with past and recent studies [11, 14, 23]. However these geographical differences seem to be decreasing.

In general, our results show significant regional and rural/semi-urban/urban variations in HIV prevalence. Therefore there is a need for national-wide scaling PMTCT services within RCH clinics. All ANC clinics should be able to provide PMTCT option B+ which require women and their infants to receive a cascade of interventions including uptake of antenatal services and HIV testing during pregnancy, use of antiretroviral treatment (ART) by pregnant women living with HIV, safe childbirth practices and appropriate infant feeding, uptake of infant HIV testing and other post-natal healthcare services [24]. The issues and challenges which have been identified to hamper the promotion of PMTCT such as stigma, male involvement, infant feeding options and decision making, home deliveries, and access to up-to-date information on PMTCT should be addressed in order to ensure uptake and utilization of PMTCT services [25, 26].

### Factors associated with syphilis infection

The only factors that were associated with risk for syphilis were living in a rural or semi-urban area and having 1 to 4 previous numbers of pregnancies. Our finding of high burden of syphilis in rural and semi-urban areas is similar to what has been reported in studies from Sub-Saharan Africa and China [12, 27–29]. The high burden of syphilis among the rural and semi-urban population in Tanzania may be partially explained by the relatively poor treatment access as well as data quality. In rural settings syphilis testing was done by ANC nurses, whom may be less skilled than laboratory technicians who performed the test in urban testing. The RPR is a macroscopic flocculation tests and requires no microscope, but has the potential for false-negative results due to prozone reactions.

For many years surveillance of syphilis in Tanzania has based on RPR, which is (non-treponemal) which are not specific and may therefore provide some false positive or negative results. We are therefore advocating use of rapid point-of-care (RPOC) tests as a new approach to ensuring that diagnosis and treatment of syphilis is timely across the diverse geographic and social settings in the country. The RPOC tests should be able to detect recent, as distinct from past treated infections.

### Association between occurrence of syphilis and HIV infections

Apparently, there was no clear correlation in the occurrence of the two infections in the different regions. For example, while the prevalence of HIV infection in Iringa region was 14.82 % that of syphilis was only 1.91 %. Similarly, the prevalence of HIV infection in Mbeya region was 11.31 %, while that of syphilis was 2.2 %. On the other hand, Mwanza region which had the highest prevalence of syphilis (6.7 %), had a rather modest prevalence of HIV infection (4.2 %). Tabora region which had syphilis prevalence of 3.07 % had a HIV prevalence of 4.74 %.

Possible explanations for lack of correlation could be part to the use of non-treponemal test for diagnosis of syphilis where my overestimate or underestimate the true case of syphilis.

The strength of our study is in Tanzania ~94 % of pregnant women make at least one antenatal care (ANC) visit [30], therefore there was a good representation data of ANC attendees which can provide a reasonable estimate of HIV and syphilis prevalence within the general population.

However, compared to population survey ANC surveillance have several limitations including is limited by geographical coverage because sampling is often not representative of smaller and more remote areas in a country, only women of certain social economic status attend the clinics, and do not provide information on women who are not pregnant or sexually active and who do not attend public health clinics and it lacks information on men [3, 31, 32]. In our study, these limitations were minimized by selecting 133 health facilities from all regions in Tanzania mainland and from each region at least 2 ANC clinics were selected from rural, semi-urban and urban areas.

Fortunately, Tanzania has opportunity of both ANC sentinel surveillance and population surveys that can complement information thereby providing clear picture of HIV infection. We therefore advocate use of both datasets for in-depth analysis of national HIV epidemic.

## Conclusions

The overall prevalence of HIV infection (5.6 %) and syphilis (2.5 %) found among pregnant women attending ANC clinics in Tanzania calls for further strengthening of current intervention measures, which include scaling up the integration of prevention of mother to child transmission (PMTCT) services in Reproductive and Child Health (RCH) clinics. The issues and challenges which have been identified to hamper the promotion of PMTCT such as stigma, male involvement, infant feeding options and decision making, home deliveries, and access to up-to-date information on PMTCT should be addressed in order to ensure uptake and utilization of PMTCT services.
